# Modelling the tumor immune microenvironment for precision immunotherapy

**DOI:** 10.1002/cti2.1400

**Published:** 2022-06-26

**Authors:** Nathan J Mackenzie, Clarissa Nicholls, Abby R Templeton, Mahasha PJ Perera, Penny L Jeffery, Kate Zimmermann, Arutha Kulasinghe, Tony J Kenna, Ian Vela, Elizabeth D Williams, Patrick B Thomas

**Affiliations:** ^1^ School of Biomedical Sciences at Translational Research Institute (TRI) Queensland University of Technology (QUT) Brisbane QLD Australia; ^2^ Queensland Bladder Cancer Initiative (QBCI) Brisbane QLD Australia; ^3^ Centre for Personalised Analysis of Cancers (CPAC) Brisbane QLD Australia; ^4^ Australian Prostate Cancer Research Centre – Queensland (APCRC‐Q) Brisbane QLD Australia; ^5^ Department of Urology Princess Alexandra Hospital Woolloongabba QLD Australia; ^6^ Centre for Immunology and Infection Control School of Biomedical Sciences Queensland University of Technology (QUT) Brisbane QLD Australia; ^7^ Centre for Microbiome Research School of Biomedical Sciences Queensland University of Technology (QUT) Brisbane QLD Australia; ^8^ University of Queensland Diamantina Institute The University of Queensland Brisbane QLD Australia

**Keywords:** co‐culture, immuno‐oncology, immunotherapy, patient‐derived explants, patient‐derived organoids, precision medicine

## Abstract

The complexity of the cellular and acellular players within the tumor microenvironment (TME) allows for significant variation in TME constitution and role in anticancer treatment response. Spatial alterations in populations of tumor cells and adjacent non‐malignant cells, including endothelial cells, fibroblasts and tissue‐infiltrating immune cells, often have a major role in determining disease progression and treatment response in cancer. Many current standard systemic antineoplastic treatments target the cancer cells and could be further refined to directly target commonly dysregulated cell populations of the TME. Recent developments in immuno‐oncology and bioengineering have created an attractive potential to model these complexities at the level of the individual patient. These developments, along with the increasing momentum in precision medicine research and application, have catalysed exciting new discoveries in understanding drug–TME interactions, target identification, and improved efficacy of therapies. While rapid progress has been made, there are still many challenges to overcome in the development of accurate *in vitro, in vivo* and *ex vivo* models incorporating the cellular interactions that take place in the TME. In this review, we describe how advances in immuno‐oncology and patient‐derived models, such as patient‐derived organoids and explant cultures, have enhanced the landscape of personalised immunotherapy prediction and treatment of solid organ malignancies. We describe and compare different immunological targets and perspectives on two‐dimensional and three‐dimensional modelling approaches that may be used to better rationalise immunotherapy use, ultimately providing a knowledge base for the integration of the autologous TME into these predictive models.

## Graphical abstract

In this article, we discuss several clinically significant immune checkpoint markers and review the current *in vitro* and *ex vivo* immune co‐culture platforms that have applications for translational precision immunotherapy in solid tumors. The figure was created with Biorender.com.

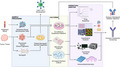

## Introduction

For decades, surgical procedures coupled with non‐targeted conventional therapies such as systemic chemotherapy and/or radiotherapy have represented standard of care (SOC) for the treatment of solid organ malignancies. However, these options lack tumor‐specific drug targets, can be costly and do not always represent the most efficacious course of treatment for an individual patient.^1^ To combat these challenges, precision medicine aims to provide ‘the right treatment for the right person at the right time in their clinical management’ by incorporating information garnered from genetic profiling to aid in the identification of optimal therapy for the individual. Despite this, there is a still an urgent need to develop additional companion diagnostic tools that can predict drug response while considering the tumor heterogeneity specific to each patient. In the last decade, improvements in personalised patient‐specific tumor models have allowed for a deeper understanding of the intricate relationship between the host immunity and the tumor microenvironment (TME), thus giving rise to an era of precision approaches to guide cancer therapy.^2^

With the approval of ipilimumab for treating metastatic melanoma in 2011,^3^ incorporation of immunotherapy regimens for the treatment of various cancers has become a viable therapeutic option in addition to conventional chemotherapies. When considering second‐line regimens in solid tumors, which progress following SOC chemotherapy, immunotherapies provide alternative mechanisms to target cancer and, in some instances, provide durable long‐term responses.^4^ Immune checkpoints are essential interactions that regulate immune responses and can be of a stimulatory or suppressive nature.^5^, ^6^ In a normal homeostatic capacity, these axes have a major protective role in preventing immunodeficiency and autoimmunity.^7^ However, many solid tumors express immune‐suppressive ligands, which are upregulated within the tumor and immune cells of TME, allowing tumor cells to evade host immune defences.^8^ Thus, the blockade of these checkpoints with monoclonal antibodies (mAbs) can restore or prolong the antitumor immune response.^8^ A small number of these mAbs have been developed for clinical use as immune checkpoint inhibitors (ICIs). However, despite durable clinical responses for subsets of melanoma, lung and bladder cancer patients, regimens of approved ICIs remain largely ineffective and difficult to predict as a monotherapy.^9^, ^10^, ^11^ Additionally, high levels of permanent and irreversible immune‐related toxicity occur in a large subset of patients.^12^ Clinical biomarkers and/or bench‐to‐bedside *in vitro* prediction tools to direct the use of many ICIs are currently lacking. Barriers in the implementation and evaluation of companion diagnostic biomarker tests that are used to stratify patients for ICIs (i.e. tumor cell expression of PD‐L1 and tumor mutation burden assessment) suggest that much remains to be discovered to fully understand ICI response. The development of rapid patient‐directed models incorporating intra‐ and inter‐patient tumor heterogeneity would allow ICI in a more targeted and rational manner, thus ameliorating the above issues and leading to more favorable and durable responses.^13^

Optimisation of personalised ICI therapy for solid tumors may be performed by utilising *ex vivo* platforms that most accurately replicate the biophysical, chemical, and cellular TME to model drug response at a patient‐specific level.^14^ Inclusive of this is the co‐culture of autologous components such as serum,^15^ and non‐neoplastic host cells (including immune cells^16^ and other stromal components such as endothelium and fibroblasts) to achieve a more representative model. Patient‐derived organoids (PDOs), resected tissue explants, bioprinting and organ‐on‐a‐chip models represent potential 3D platforms, which more faithfully recapitulate the TME than current 2D methods do.^14^ Such developments have shown promise but are still in their infancy. Besides estimating checkpoint biomarker expression, clinicians lack standardised assays that can be used to inform treatment decisions. Thus, paramount to the success of these models is the understanding of key functional immune components in tumorigenesis. Herein, we discuss whether the concept of a 3D immuno‐oncological model inclusive of autologous stromal and/or immune interactions is a feasible and practical approach to predict immunotherapy response to guide precision treatment strategies in the context of the current understanding of the composition and modelling of the immune TME.

## Immune cells of the tumor microenvironment

**Figure 1:**
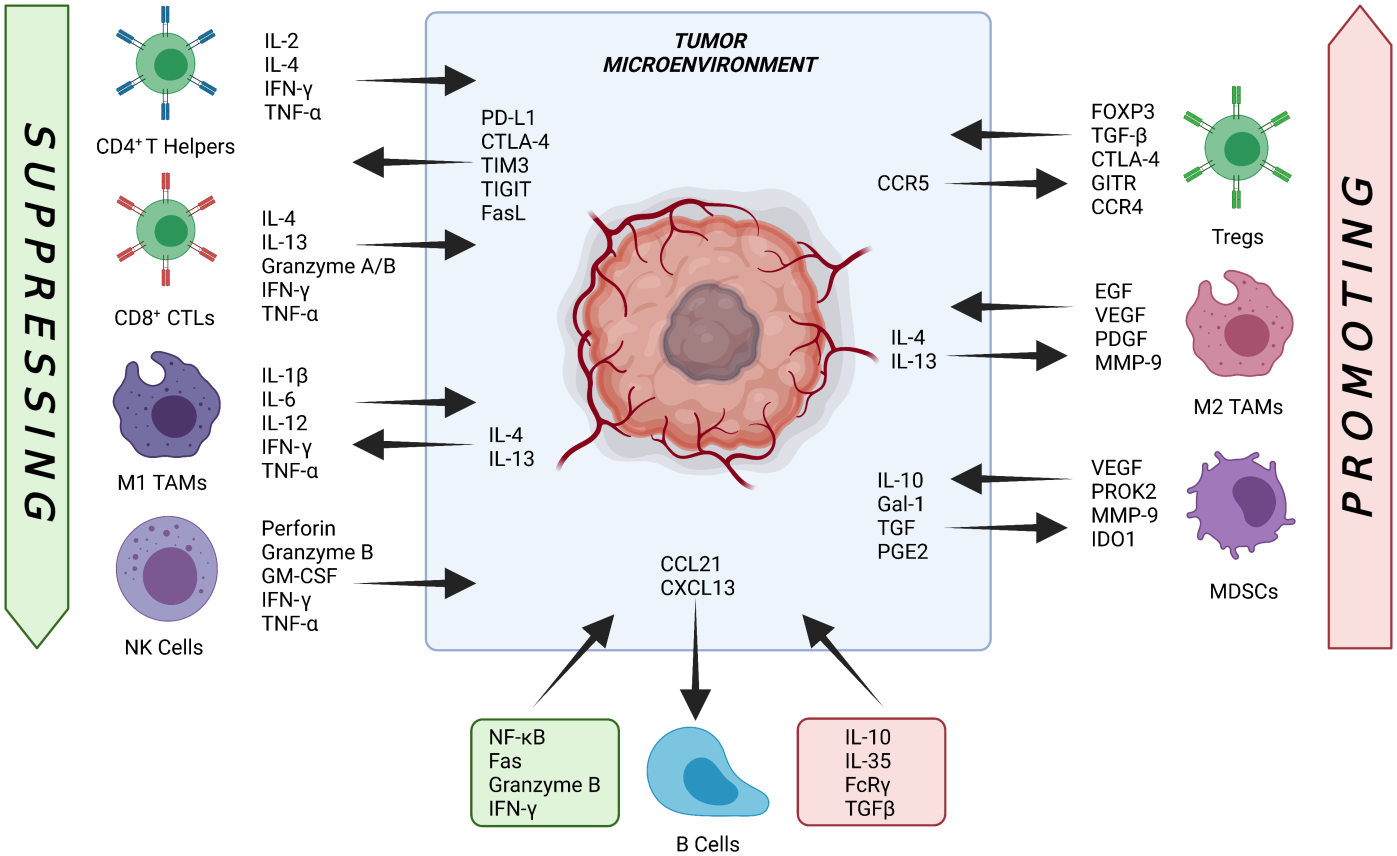
Cellular immunome of the tumor microenvironment. Solid tumors establish both protumoral and immunosuppressive microenvironments comprising complex combinations of various tumor‐derived soluble factors and cytokines to sustain growth, support tumorigenesis and dormancy and promote immune evasion mechanisms. Core to this is the cellular immune components of the tumor microenvironment (TME), including intact, highly activated T‐helper cells, cytotoxic T lymphocytes (CTLs), M1 tumor‐associated macrophages (TAMs) and natural killer (NK) subsets. Following the initiation of oncogenesis, immunological rejection of tumors is largely mediated by tumor‐infiltrating T cells. Chronic activation causes upregulation of exhaustion‐associated molecules, including programmed death‐ligand 1 (PD‐L1), cytotoxic T‐lymphocyte‐associated antigen 4 (CTLA‐4) and T‐cell immunoglobulin and mucin domain‐containing protein 3 (TIM3). This figure was created with Biorender.com.

The TME is manifested by cellular and acellular tumor–host interactions and has a role in governing growth, metastasis, host evasion and drug response.^17^ The TME is composed chiefly of the stroma, angiogenic factors and the inhibitory and stimulatory signals produced by infiltrating immune cells involved in innate and adaptive immunity^18^, ^19^ (summarised in Figure 1). Additional cellular components include the tumor‐associated fibroblasts, adipocytes and angiogenesis‐promoting endothelial cells.^17^ Other contributing TME factors include a range of interstitial fluid pressures, shifting hypoxia gradients, metabolic factors, nutrient starvation, immunosuppressive cytokines, upregulated immune checkpoints and mechanical stresses.^17^ During tumorigenesis, the TME is disrupted such that the cell–cell and cell–stroma interactions are altered to activate new signalling pathways, neovascularisation and dysregulated cell death resistance mechanisms.^17^ Immunological changes affect not only tumor‐infiltrating lymphocytes (TILs) but also circulating immune cells such as peripheral blood mononuclear cells (PBMCs).^20^ Broadly, there are two main categories of tumor‐associated immune cells: tumor‐suppressing or tumor‐promoting cells.^21^

### Tumor‐suppressing cells

During tumor cell elimination, effector T cells, including CD4^+^ helper (T_h_) and CD8^+^ cytotoxic lymphocytes (CTLs), secrete cytokines IFN‐γ and TNF‐α to induce tumor cell death and recruit other effector cells.^22^ In addition, activated T_h_ cells produce the T‐cell growth factor IL‐2.^22^ Natural killer (NK) cells are recruited to tumor cells via identification of downregulated or altered MHC class I molecules.^23^ Additionally, CTLs and NK cells also secrete IFN‐γ and TNF‐α, which further potentiate apoptotic effects through the release of granzymes and perforin.^23^ IFN‐γ and TNF‐α, alongside stimulatory factors such as IL‐6 and GM‐CSF, promote an inflammatory response in the TME.^24^ CTLs, T_h_ and NK cells, along with phagocytic M1 ‘classically activated’ tumor‐associated macrophages (TAMs) and N1‐polarised neutrophils, and pro‐inflammatory dendritic cells (DCs) make up the majority of tumor‐suppressing immune cells present in the TME.^25^

### Tumor‐promoting cells

In a state of favorable vascularisation, stromal composition and chemotactic signalling, the TME can recruit or induce cells into a state of tumor promotion.^6^ Regulatory T cells (Tregs) represent an immunosuppressive subset of T_h_ cells.^26^ Identified by detectable levels of membrane‐bound CD25 and transcription factor forkhead box P3 (FOXP3), Tregs have an important immunosuppressive role in mediating immune self‐tolerance and resolving inflammation.^27^ Tregs perform this role chiefly by the production of inhibitory molecules, and sequestering of IL‐2‐ and cytotoxic T‐lymphocyte antigen 4 (CTLA‐4)‐mediated suppression of antigen‐presenting cells (APCs).^26^ However, within the TME this immunosuppressive action becomes problematic, acting principally on effector T cells and thereby disabling their antitumorigenic effects.^26^ Additionally, by inducing oxidative stress *via* the depletion of amino acids, suppressing recruitment of effector cells and promoting recruitment of regulatory cells, myeloid‐derived suppressor cells (MDSCs) infiltrating in the TME can disrupt the efficiency of host antitumor immunity and increase metastatic potential.^28^ MDSCs also promote tumorigenesis through the induction of angiogenesis through secretion of proangiogenic factors such as vascular endothelial growth factor, matrix metalloproteinase 9 and prokinectin‐2.^29^ Studies in both murine and human cell models have also shown that increased circulating levels of cancer‐associated MDSCs reduce ICI therapy efficacy, most likely because of the general suppression of T‐cell function and recognition.^30^, ^31^ M2 (or ‘alternatively activated’) macrophages are a class of macrophage that unlike the M1 subset contribute to tissue repair and fibrosis *via* induction of immune suppression and an anti‐inflammatory response.^29^

## Immune checkpoints of the tumor microenvironment

**Figure 2:**
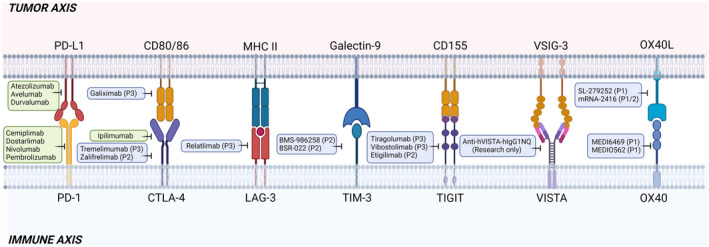
Immune checkpoints at the tumor–immune axis displaying checkpoint ligands and clinically relevant inhibitors. Inhibitors with full United States Food and Drug Administration (FDA) approval are indicated in green boxes. BMS, Bristol Meyers Squibb; BSR, British Society for Rheumatology; CD, cluster of differentiation; CTLA‐4, cytotoxic T‐lymphocyte‐associated protein 4; LAG‐3, lymphocyte activation gene 3; MHC II, human major histocompatibility complex II; OX40, tumor necrosis factor receptor superfamily, member 4; OX40L, OX40 ligand; P2, Phase II clinical trial; P3, Phase III clinical trial; PD‐1, programmed death‐1; PD‐L1, programmed death‐ligand 1; TIGIT, T‐cell immunoreceptor with Ig and ITIM domains; TIM‐3, T‐cell immunoglobulin and mucin domain‐containing 3; VISTA, V‐domain immunoglobulin suppressor of T‐cell activation; VSIG‐3, V‐set and immunoglobulin domain‐containing 3. This figure was created with Biorender.com.

Immune checkpoint inhibitors (ICIs) are arguably the most significant development in cancer therapy over the past decade and function as key inhibitory signalling molecules to attenuate the robust antitumor immune responses in the TME.^32^ ICIs are expressed on a wide range of cells and act throughout the early phase of immune activation and throughout the ongoing response to impair T‐cell activation.^33^ The most widely investigated ICIs currently in immuno‐oncology are PD‐1 (CD279), PD‐L1 (CD274, B7‐H1) and CTLA‐4 (CD152). Immune checkpoints are widely expressed on the surface of APCs, tumor cells and TILs and function as immunosuppressive receptors to induce CTL exhaustion and anergy.^34^ Others, including T‐cell immunoglobulin and mucin domain 3 (TIM‐3), lymphocyte activation gene 3 (LAG‐3), T‐cell immunoreceptor with Ig and ITIM domains (TIGIT), V‐domain Ig suppressor of T‐cell activation (VISTA), and co‐stimulatory molecules such as the TNF receptor/TNF superfamily members OX40 (CD134) and OX40 ligand (OX40L; CD252), are emerging clinical drug targets in several clinical settings (Figure 2). Approximately two thirds of all oncology trials are dedicated to T‐cell‐targeting immunomodulators (Table 1), and there are more than 3000 ongoing clinical trials (https://clinicaltrials.gov). CTLA‐4 and PD‐1/PD‐L1 in particular have been found to be highly expressed in a wide range of malignancies and have subsequently attracted the most interest to date.^35^ Currently, there are a handful of FDA‐approved ICIs targeting the CTLA‐4 and PD‐1/PD‐L1 axes in various cancers, with several novel mAbs currently being explored for clinical efficacy (Figure 2, Table 1). While ICIs provide long‐lasting efficacy for a subset of patients, a large majority do not respond to treatment and experience adverse side effects.^36^ An urgent clinical need necessitates the development of novel predictive biomarkers and personalised ICI testing in patient‐derived tumor cells. Below, we review current and novel immune targets that are emerging in clinical and preclinical research.

**Table 1:** Existing immune checkpoint inhibitor trials and emerging targets in solid tumors

Target	Inhibitor (commercial name)	Trial name	Company	ClinicalTrials.gov Identifier	Trial phase	Trial stage	Malignancy	Clinical setting
CTLA‐4	Ipilimumab (Yervoy)	COSINR	Bristol Myers Squibb	NCT03223155	I	Active, recruiting	Stage IV NSCLC	nivolumab/ipilimumab plus either sequential or concurrent SBRT
		/		NCT01688492	I/II	Active, not recruiting	MCRPC	Chemotherapy‐ and immunotherapy‐naïve progressive mCRPC
		CheckMate 401		NCT02599402	III	Complete	Stage III melanoma	Adjunct with nivo for treatment‐naïve melanoma
	Tremelimumab	/	MedImmune (AstraZeneca)	NCT01843374	IIb	Active, not recruiting	Mesothelioma	Unresectable mesothelioma following first‐line platinum‐based regimen
		/		NCT03557918	II	Active, recruiting	mUC	Post‐anti‐PD‐1/PD‐L1 therapy with no improvement
	Zalifrelimab	NUMANTIA	Agenus	NCT04827953	Ib/IIa	Recruiting	PDAC	Following first‐line SOC chemotherapy
LAG‐3	Relatlimab (BMS‐986016)	RELATIVITY	Bristol Myers Squibb	NCT03470922	II/III	Active, not recruiting	Advanced melanoma	Pretreatment; adjunct with nivo
				NCT04567615	II	Active, recruiting	HCC	Adjunct with nivolumab; post‐TKI treatment; IO therapy naïve
PD‐1	Cemiplimab (Libtayo)	EMPOWER ‐Lung 1	Regeneron Pharmaceuticals	NCT03088540	III	Active, not recruiting	NSCLC	> 50% PD‐L1 expression, no EGFR, ALK, ROS1 mutations
	Nivolumab (Opdivo)	CheckMate649	Bristol Myers Squibb	NCT02872116	III	Active, not recruiting	Gastric/GEJ, EAC	CPS ≥ 5%, HER2‐neg, in combination with SOC chemotherapy
	Pembrolizumab (Keytruda)	KEYNOTE‐630	Merck	NCT03833167	III	Active, recruiting	cSCC	Post‐surgery, post‐radiation
		KEYNOTE‐672/ECHO‐307		NCT03361865	III	Complete	mUC	Cis. ineligible, adjunct with epacadostat *vs* placebo
PD‐L1	Atezolizumab (Tecentriq)	BIRCH	Hoffmann‐La Roche	NCT02031458	II	Complete	(Stage IIIB, Stage IV, or recurrent) NSCLC	PD‐L1 pos tumors
		OAK		NCT02008227	III	Complete		
		POPLAR		NCT01903993	III	Complete		
	Avelumab (Bavencio)	JAVELIN Bladder 100	Pfizer	NCT02603432	III	Active, not recruiting	Stage IV mUC	Maintenance following first‐line platinum‐based SOC
	Durvalumab (Imfinzi)	CASPIAN	AstraZeneca	NCT03043872	III	Active, not recruiting	SCLC	Treatment naïve with platinum–etoposide/tremelimumab
		PACIFIC		NCT02125461	III	Active, not recruiting	NSCLC	Following SOC chemotherapy, unrespectable cancer
TIGIT	Tiragolumab	SKYSCRAPER‐02	Genentech	NCT04256421	III	Active, not recruiting	SCLC	Combination atezo + CE
	Vibostolimab (MK‐7684)	MK‐7684‐001	Merck	NCT02964013	I	Recruiting	Advanced solid tumors	In combination with pembro
	Etigilimab (OMP‐313 M32)	/	Mereo BioPharma	NCT04761198	I/II	Recruiting	Advanced solid tumors	Undergoing evaluation with nivo
TIM‐3	Sabatolimab (MBG453)	/	Novartis	NCT02608268	I/II	Active, not recruiting	Advanced solid tumors	Progression despite SOC, immunotherapy naïve
	Cobolimab (TSR‐022)	AMBER	GlaxoSmithKline (ex‐Tesaro)	NCT02817633	I	Recruiting	Advanced solid tumors	First‐in‐human dose escalation and dose expansion
OX40L	SL‐279252 (PD1‐Fc‐OX40L)	/	Shattuck Labs, Inc.	NCT03894618	I	Recruiting	Advanced solid tumors or lymphomas	First‐in‐human dose escalation and dose expansion
	mRNA‐2416	/	ModernaTX, Inc.	NCT03323398	I/II	Active, not recruiting	Relapsed/refractory solid tumor malignancies or lymphoma	In combination with durvalumab
OX40	MEDI0562	/	MedImmune (AstraZeneca)	NCT02705482	I	Complete	Advanced solid tumors	First‐in‐human dose escalation and dose expansion in combination with durvalumab and tremelimumab
	MEDI0562	/	MedImmune (AstraZeneca)	NCT03336606	Ib	Active, not recruiting	Advanced HNSCC or stage IIIb/IIIC melanoma	Single agent in the preoperative setting
	MEDI6469	/	MedImmune (AstraZeneca)	NCT02274155	Ib	Active, not recruiting	HNSCC	Stage III and IV HNSCC. Safety and feasibility of preoperative administration

ALK, anaplastic lymphoma kinase; atezo, atezolizumab; CE, carboplatin and etoposide; Cis, cisplatin; CPS, combined positive score (PD‐L1^+^ cells (tumor cells, lymphocytes, macrophages)/total number of viable tumor cells × 100; cSCC, cutaneous squamous cell carcinoma; EAC, oesophageal adenocarcinoma; EGFR, epidermal growth factor receptor; GEJ, oesophagogastric junction; HCC, hepatocellular carcinoma; HER2, human epidermal growth factor receptor 2; HNSCC, head and neck squamous cell carcinoma; IO, immuno‐oncology; mCRCP, metastatic castration‐resistant prostate cancer; mUC, metastatic urothelial carcinoma; nivo, nivolumab; NSCLC, non‐small‐cell lung cancer; PDAC, pancreatic adenocarcinoma; PD‐L1, programmed death‐ligand 1; pembro, pembrolizumab; ROS1, ROS proto‐oncogene tyrosine‐protein kinase; SBRT, stereotactic body radiotherapy; SCLC, small‐cell lung cancer, SOC, standard of care; and TKI, tyrosine kinase inhibitor.

## CTLA‐4

CTLA‐4 is a B7/CD28 family membrane glycoprotein constitutively expressed on T cells and is a potent negative regulator of the antitumor T‐cell response.^37^ Following TCR activation, CD28 on the cell surface of T cells binds to CD80 (B7‐1) or CD86 (B7‐2) on APCs, resulting in T‐cell maturation and subsequent immune function.^37^ CTLA‐4, being structurally homologous to CD28, competes for CD80 at a higher affinity when trafficked to the cell membrane from cytoplasmic microvesicles, impairing IL‐2 production and preventing T‐cell maturation and causing immune suppression.^37^ Treg cells constitutively express CTLA‐4, which acts to promote immunosuppression in the TME.^26^ The first‐in‐class monoclonal anti‐CTLA‐4 antibody ipilimumab (Yervoy; Bristol Myers Squibb) was the first ICI to gain FDA approval^38^ and has been shown to induce durable responses, allowing a subset (16–20%) of advanced stage IV melanoma patients to achieve stable disease.^39^

## PD‐1/PD‐L1

Shortly after the discovery of CTLA‐4, Honjo *et al*.^40^ identified a 288 amino acid type I transmembrane apoptosis‐mediating protein, which they characterised as programmed death‐1. The binding of PD‐1 on T, B and NK cells to its ligand, PD‐L1/PD‐L2, found most commonly on DCs, epithelial cells and macrophages, lowers the threshold for apoptosis and ultimately results in T‐cell depletion through T‐cell receptor (TCR)‐mediated PI3K/Akt/Ras–MEK/ERK signalling pathways.^41^ PD‐L1 is detectable on the surface of many tumor types, including and not limited to: colorectal, bladder, breast, melanoma and lung cancers.^42^ Upregulation of this axis has been implicated in many malignancies and is perhaps the most well‐studied immune checkpoint.^43^, ^44^ Owing to increased interest in the field, several immune checkpoints have been uncovered through revisiting the PD‐1/PD‐L1 axis in relation to other regulatory pathways. To date, approximately seven PD‐1/PD‐L1‐targeting drugs are approved for over nine cancers (Figure 2), with many other inhibitors currently under development and or in clinical trials (> 1500 at current standing; https://clinicaltrials.gov). Both anti‐PD‐L1 agents, durvalumab (Imfinzi; Medimmune/AstraZeneca), avelumab (Bavencio; Merck & Pfizer) and atezolizumab (Tecentriq; Genentech), and anti‐PD‐1‐targeting agents, nivolumab (Opdivo; Bristol Myers Squibb) and pembrolizumab (Keytruda; Merck Sharp & Dohme Corp), have shown durable responses and paved the way for treatment of many unresectable, mismatch repair‐deficient (dMMR)/microsatellite instability‐high (MSI‐H) tumors, or cancers that have progressed following first‐line platinum‐based chemotherapy or chemoradiation regimens (in the case of non‐small‐cell lung carcinoma (NSCLC) with PD‐L1 expression ≥ 1%).^45^, ^46^

## TIM‐3

TIM‐3 is widely expressed and can be detected on CTLs, Tregs, B‐cells NK cells, DCs, TAMs and tumor cells^47^ and promotes T‐cell exhaustion through antagonism of TCR signalling and expansion of MDSCs within the immunosuppressed TME.^24^ The TIM‐3/Gal‐9 axis is the most widely studied and is known to induce apoptosis through Ca^2+^ influx.^48^ Thus, the blockade of this pathway can result in restored immune functionality in the TME. TIM‐3 is distinctly perturbed among the CD8^+^PD1^+^ T and Treg cells within the tumor TME,^48^ and the blockade in a preclinical setting has been found to be effective in models of pancreatic cancer, colorectal cancer and NSCLC.^49^ It has been previously demonstrated that resistance to anti‐PD‐1/PD‐L1 treatment correlated with an adaptive upregulation of alternative immune checkpoints, including TIM‐3.^50^ Consequently, promising results have been seen with anti‐TIM‐3 mAbs used in combination with anti‐PD‐1/PD‐L1 mAbs and oncolytic viral therapy in lung cancer.^51^ Approximately 15 companies are developing TIM‐3‐targeting antibodies, with Novartis (sabatolimab) the only one expecting Phase III trial outcomes in early 2027 in the clinical setting of high‐risk myelodysplastic syndrome and chronic myelomonocytic leukaemia‐2 (STIMULUS‐MDS2; NCT04266301).

## LAG‐3

LAG‐3 (CD223) is structurally homologous to CD4 and is expressed on peripheral Tregs, CTLs, B cells, NK cells and DCs.^52^ LAG‐3 is also highly expressed on CD8^+^ TILs, and it has been proposed that LAG‐3 may be used as an indicator of tumor prognosis and as a target for ICI therapy.^53^ While the exact mechanism of LAG‐3 is to be fully elucidated, studies show that this molecule interacts with major histocompatibility complex (MHC) class II on APCs at a higher affinity than CD4 (~40 times higher) and suppresses T‐cell activation and cytokine production, maturation and expansion.^53^ Conversely, it is also expressed by plasmacytoid DCs, which in turn express many of the immune checkpoints that exhausted T cells interact with.^54^ Notably, dual blockage of LAG‐3 (using relatlimab) and PD‐1 (using nivolumab) was shown to have effective synergism and higher clinical efficacy in a subset of melanoma patients with progressive disease who previously showed no durable response to anti‐PD‐1/PD‐L1 monotherapy.^55^ However, further randomised, double‐blinded Phase II/III trials are required to assess whether combining LAG‐3 blockade with other ICIs leads to increased incidence and severity of irAEs.

## TIGIT

TIGIT is a next‐generation emerging target in immune checkpoint therapy. Expressed predominantly on activated CTLs, Tregs and NK cells, TIGIT binds to either poliovirus PVR or its homologue nectin‐2 expressed on APCs and tumor cells to attenuate T‐cell‐mediated pattern recognition and NK‐mediated cytotoxicity.^56^ Expression of TIGIT has since been observed in NSCLC, urothelial, melanoma, gastric, colorectal, breast and prostate cancers.^43^, ^57^ Not unlike TIM‐3, TIGIT has also been found in many cases to be adaptively upregulated in patients treated with anti‐PD‐1/PD‐L1 and is potent enough to abrogate CD8^+^ T‐cell and NK cell cytotoxicity.^58^ *In vivo*, this occurs through the suppression of CD8^+^ T‐cell cytotoxicity *via* IL‐10 production.^59^ TIGIT^+^ Tregs, when compared to Tregs lacking expression, suppress the function of Th1 inflammatory cell types through distorting CD4^+^ T‐cell response towards a Th2 profile.^59^ Favorable results have been achieved in a preclinical setting using a co‐therapy of anti‐PD‐1/PD‐L1 and anti‐TIGIT.^58^, ^60^ The anti‐TIGIT mAb tiragolumab is currently in Phase III clinical trials in combination with atezolizumab and carboplatin/etoposide in chemotherapy‐naïve small‐cell lung cancer (SKYSCRAPER‐02; NCT04256421) (Table 1). Alongside these trials, randomised investigation into tiragolumab and atezolizumab combinations in the clinical setting of early‐stage NSCLC (SKYSCRAPER‐03; NCT04513925), advanced oesophageal cancer (SKYSCRAPER‐07; NCT04543617) and metastatic oesophageal cancer (SKYSCRAPER‐08; NCT04540211) is currently recruiting.

## VISTA

Currently, VISTA represents one of the more recent immune checkpoints implicated in infiltrated–inflamed TMEs.^25^ Preliminary studies indicate VISTA negatively mediates immune cell quiescence,^61^ achieved principally by interaction with V‐set and immunoglobulin domain‐containing 3 (VSIG‐3). In the excessively acidic environment caused by lactic acid production within the TME, VISTA selectively binds with PSGL‐1 to suppress T‐cell activity.^62^ Recent reports show that VISTA is predominantly expressed on myeloid lineage cells, Tregs and naïve T_h_ cells – where its potential as an immunotherapy target is becoming increasingly appreciated.^63^ Additionally, VISTA is also expressed, albeit to a lesser extent, on monocytes, neutrophils and NK cells.^61^ VISTA has been documented to be highly overexpressed in some genitourinary cancers and high‐grade serous ovarian cancer, when compared to normal tissue.^64^, ^65^ Mulati *et al*.^63^ discovered that by generating VISTA‐knockdown variants of human ovarian and endometrial cancer cell lines (COV504 and HEC1A, respectively), T‐cell proliferation and cytokine secretion were markedly restored. The development of pH‐selective VISTA antibodies in immune checkpoint‐based cancer therapy is currently still unknown; however, anti‐VISTA mAbs (e.g. the novel SNS‐101 anti‐VISTA mAb; Sensei Biotherapeutics, Inc.) may potentiate anti‐PD‐1/PD‐L1‐induced antitumor immunity. Given its important role in regulating innate and adaptive immune responses, VISTA is a promising target for immunotherapeutic intervention.

## Immune‐activating checkpoints

### OX40/OX40L axis

The OX40/OX40L co‐stimulatory axis represents the next generation of immunotherapies, which are aimed at reshaping the antitumor immune TME. OX40L is expressed on many APCs such as DCs, macrophages and activated B cells but is also expressed on tumor cells, endothelial cells and smooth muscle cells.^66^ Its binding to OX40 serves as a co‐stimulatory signal to induce cytokine production, T‐cell survival, clonal division and development of memory T cells.^66^ OX40/OX40L signalling has been shown to abolish Treg immunosuppressive functions, reduce Foxp3 expression and promote the proliferation of CD4^+^ and CD8^+^ T lymphocytes.^67^ Like CTLA‐4, OX40 is upregulated in TILs when compared to its very low basal expression in peripheral blood lymphocytes.^66^ The literature regarding the expression of OX40/OX40L in tumors is still relatively unclear. Kashima *et al*.^68^ determined that high serum levels of OX40 and OX40L in NSCLC were associated with poor prognosis, although high levels of OX40L were associated with improved survival following treatments with ICIs. In the TME, secreted OX40L from cancer‐associated fibroblasts (CAFs) under stress conditions can facilitate cisplatin resistance and inhibition of apoptosis of lung adenocarcinoma cells through activation of NF‐κB/BCL‐XL pathways.^69^ However, other reports indicate that in different contexts, high OX40 expression is associated with increased CD4^+^ TIL infiltration and a favorable immune profile.^70^, ^71^ In oral squamous cell carcinoma (OSCC) and hepatocellular carcinoma, high OX40 is associated with advanced disease,^70^, ^71^ possibly reflecting the immune‐exhausted, immunosuppressive TME. Similarly, Lecerf *et al*.^72^ showed that approximately 85% of head and neck squamous cell carcinoma (HNSCC) tumors showed high expression levels of *OX40*/*OX40L*, where *OX40L* mRNA expression in the context of low *PD‐1* expression was associated with high recurrence rates. Both *OX40* and *OX40L* are overexpressed in muscle invasive bladder tumors, where the risk of recurrence in non‐muscle invasive bladder cancer is reported to be approximately twofold higher with *OX40L* mRNA overexpression.^73^ Clinically, humanised monoclonal agonists targeting the OX40/OX40L axis have shown promise by decreasing intratumoral OX40^+^ FOXP3^+^ cells to enhance adaptive immunity in the solid tumor TME.^74^ Combining conventional immune checkpoint inhibition of CTLA‐4 and PD‐1 may enhance the Treg depletion within the tumor and further potentiate the CD4^+^ and CD8^+^ antitumor immunity (Table 1).

### *In vitro/ex vivo* replication and preservation of the TME

**Figure 3:**
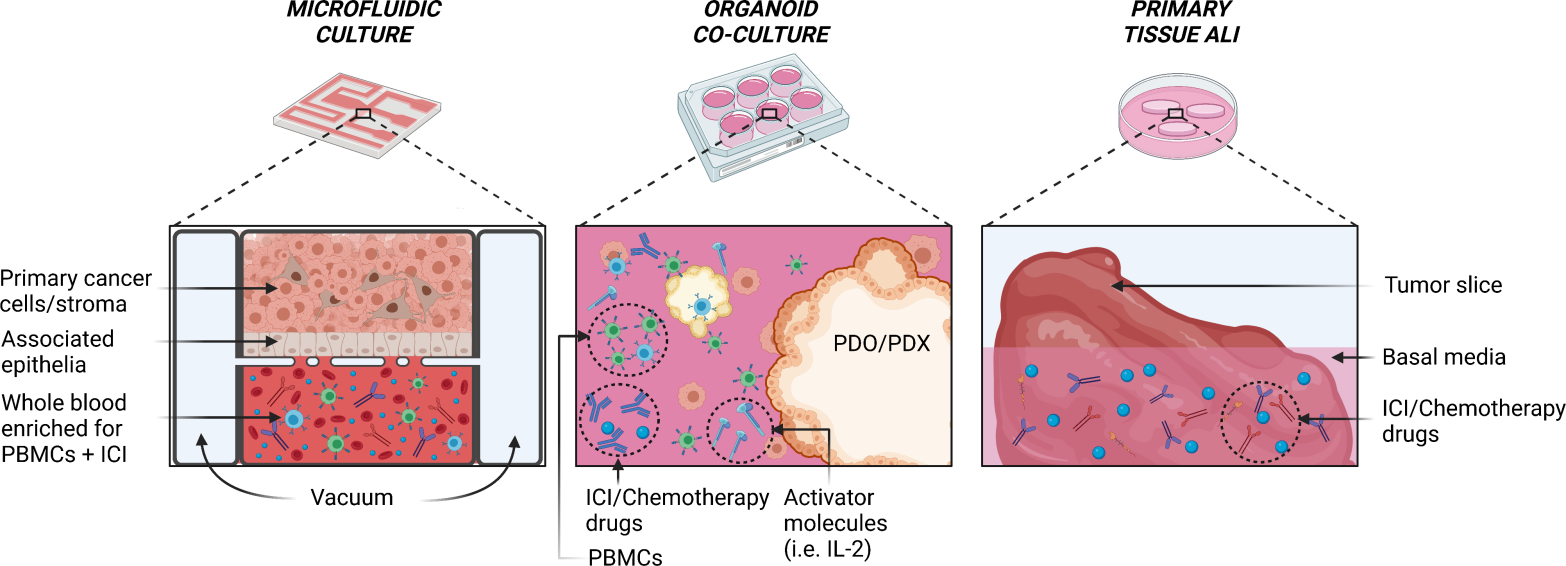
Representation of patient‐derived tumor–immune co‐cultures. A cross section of a microfluidic chamber is displayed, showing primary tumor cells injected into a chamber with whole blood in the adjacent chamber. Pores between these chambers facilitate movement of material across the epithelium. Free floating, or suspended PDOs and PDXs are commonly cultured in an organoid medium with chemo‐ and immunotherapeutic drugs and immune cell activator molecules such as IL‐2. Alternatively, tissue slices or explants may be directly cultured in a basal media such as RPMI alongside chemo‐ and immunotherapeutic drugs to create an air–liquid interface, like what is the case in many organ niches. ALI, air–liquid interface; ICI, immune checkpoint inhibitor; IL‐2, interleukin‐2; PBMCs, peripheral blood mononuclear cells; and PDO, patient‐derived organoid. This figure was created with Biorender.com.

The success of a prescribed immunotherapy relies on an understanding of the complex network of immune evasion mechanisms intrinsic to the TME. Although ICIs are increasingly central to regimens prescribed in advanced recurrent solid tumors, our understanding of the rational use of the various therapeutic options to provide the best clinical outcome is still lacking. Additionally, only a small subset of patients has a meaningful clinical response to ICIs and some tumor types remain completely refractory or develop treatment resistance. This leads to one of the greatest challenges in the field of precision immunotherapy, which is the development of immune‐intact, robust, reproducible and cost‐effective immunotherapy testing platforms to model treatment responses. Such a platform should be one that incorporates cells homologous to the host TME, models the immunosuppressive features of the TME and retains cellular viability over drug treatment duration. Advances in biotechnology have made patient material more accessible through patient‐derived organoid (PDO) generation and tumor tissue in explant cultures. Despite well‐established and novel immunotherapies being approved and employed in the treatment of various solid tumors, *in vitro* and *ex vivo* models may be able to replicate patients' immune TME, predict personalised responses to ICIs and provide predictive information on the safety of immunotherapies (current models are shown in Figure 3 and summarised in Table 2). In the last few years, there has been progress in developing microphysiological *in vitro* systems to tease apart preclinical immune responses. Here, we summarise the major models used to investigate ICI responses, including their advantages and disadvantages and incorporation of TME elements.

**Table 2:** Comparison of major patient‐derived model co‐culture techniques for personalised immunotherapy drug treatment

	Submerged organoid/spheroid co‐cultures	3D microfluidic cultures	Primary tissue cultures
Tissue processing	Physical and enzymatic dissociation of tissue followed by filtration for 100‐μm fragments and generation of organoids in ULA plates	Standard preparation of PDOs, which are then embedded into ECM‐based biomaterial Primary cells are processed into bioinks for bioprinting	Tissue is sliced at a thickness of 100–400 μm or physically minced into small fragments
Culture Preparation	Free‐floating organoids in an ALI or suspended in a suspension medium (e.g. containing Matrigel™ or Happy Cell®) Autologous PBMCs, native TILs and anticancer drugs embedded into suspension	PDOs in collagen are injected into a 3D microfluidic culture device Primary tumor bioinks are laid onto surface of microfluidic chamber Whole blood enriched for PBMCs with the addition of anticancer drugs injected into adjacent chambers	Tumor slices or fragments directly cultured with anticancer drugs in a free‐floating or suspended setting Tumor slices embedded onto a vehicle such as a scaffold, gel or sponge
Advantages	Relatively easy expansion and enrichment of PDOs; displays phenotypic changes of the source tumor Can be used to assess drug efficacy Autologous components such as PBMCs are easily co‐cultured Representation of tumor–TME interactions	Requires only a small number of cells, media and reagents per test enables the study of tumor–immune interactions; faithful recapitulation of the TME Immune cells can be added to assess infiltration kinetics Can be highly reproducible and imaged in real time	Accounts for tumor heterogeneity across the tissue Preserves diverse population of immune cells, fibroblasts and stroma in TME Allows immune TME of primary tissues to be reconstituted
Limitations	Native stromal and immune components may not be present Short cultivation periods No standard protocols If Matrigel™ is used: Lot‐to‐lot variation and mouse origin can make it sometimes unsuitable for human studies	Small‐scale application only Process requires specialised equipment. Currently limited standardisation for 3D bioprinters Long‐term culturing has not been reported No standard protocols	Difficulty in precisely regulating fragment and slice size Viability of primary tissue is highly variable Lack of uniformity in the composition of slices. Immune cell and fibroblast count declines over 1–2 months No standard protocols
Potential in studying the tumor–immune axis	Co‐culture of autologous PDOs and PBMCs enriches tumor‐reactive T cells, which can be used to assess the efficiency of T‐cell‐mediated cytotoxicity Enables assessment of tumor organoid killing by co‐culture with TILs.	Recapitulates response to ICIs Useful culture system to test therapeutic combinations to enhance response Useful in evaluation of drug toxicity and interaction	Functional T‐cell activation and responses to ICI antibodies are faithfully preserved Conservation of TCR expression in tissue slices and fragments for accurate immune–tumor interaction Cytokine profiling through conditioned media is also possible

ALI, air–liquid interface; ECM, extracellular matrix; ICI, immune checkpoint inhibitor; PBMC, peripheral blood mononuclear cell; PDO, patient‐derived organoid; TCR, T‐cell receptor; TIL, tumor‐infiltrating lymphocyte; TME, tumor microenvironment; and ULA, ultra‐low attachment.

## Co‐culture models for predicting immunotherapy response

### Cell line models of cancer

Immortalised cell lines derived from human cancers remain one of the most standard and well‐established preclinical tools for the evaluation of cancer cell activity and drug efficacy.^75^ Cell lines are relatively low cost, common and easily manipulated to yield large supplies of experimental material, can be used to identify novel drugs and are easily imaged using standard microscopy techniques.^76^ 2D cultures are subject to relatively uniform medium perfusion, and thus are exposed to supraphysiological levels of growth factors, nutrients and oxygen, which need to be physically replaced every few days. As such, few 2D models are employed to tease apart preclinical response to immune‐targeting agents. Employing a simple 2D immune cell co‐culture to model immunosuppression, Zheng *et al*.^77^ demonstrated that co‐culture of breast cancer cell lines with anti‐CD3/CD28‐stimulated healthy donor PBMCs significantly decreased T‐cell activity as indicated by reduced INF‐γ and IL‐2 secretion in cells with high PD‐L1 levels. Flexible label‐free biosensor systems such as the xCELLigence system have been used to provide real‐time quantitative assays to monitor effector cell:target cell interactions. Cerignoli *et al*.^78^ developed a real‐time potency assay employing sensitive impedance measurements to directly investigate PC‐3 prostate cancer cell cytotoxicity changes when co‐cultured with healthy donor PBMCs. Demonstrating the utility of this model to quantitively assess immunotherapeutic approaches, anti‐PD‐1 treatment in combination with prestimulated PBMCs showed increased PC‐3 cytotoxicity.^78^ However, because of a poor recapitulation of the TME and cellular drug response, the aforementioned approaches hold limited potential as a tool to provide efficient patient‐derived *in vitro* model screening. While primary cells can be developed from the patient's tumor, the field is moving away from this method of culture as this application requires extensive lineage testing, considerable time and materials. Given their reproducibility and ease of establishment, 2D co‐cultures hold most utility during the research and development phases of immunotherapeutic approaches.

### Patient‐derived organoids

The increasingly widespread adoption of three‐dimensional (3D) cell line models (spheroids) has been considered an important improvement from conventional 2D cultures. Subsequently, PDO technology has gained popularity as an effective and rapid 3D tool that better retains the tumor‐specific genetic and molecular diversity of the original host.^79^, ^80^ They differ from established cell line spheroids by the retention of multiple organ‐specific cellular populations and stem cell components to form more complex and personalised milieu that more closely simulates the solid tumor mass.^81^, ^82^ Compared to 2D and 3D cell line‐based spheroids, PDOs better reproduce many tumor features, including hypoxia, nutrient diffusion, metabolism (i.e. decreased glucose uptake) and the eventual formation of an immunosuppressive central necrotic core.^83^ Additionally, PDOs offer more efficient cell–cell and cell–matrix interactions, influencing cell phenotype, signalling pathways, adhesion and overall mechanosensing.^81^ PDOs are compatible with high‐throughput assays that in turn rapidly provide quantifiable and clinically relevant data.^84^ Additionally, PDOs can be established from a small sample of tissue such as that obtained in a fine‐needle biopsy, allowing potential generation from small cancers, or those in which sampling is limited.^80^

Patient‐derived organoids that can retain host‐derived immune cell subsets and organoid‐based propagation *en bloc* with immune stroma are a valuable candidate for holistic approaches to 3D immuno‐oncology TME modelling. However, these models historically are prohibited by host‐derived immune cells rapidly losing viability before studies can explore patient responses. Pancreatic ductal adenocarcinoma PDOs cultured with autologous PBMCs and CAFs have been found to retain certain elements of stromal influence in the TME.^85^ Replicative of the native TME, activation of myofibroblast‐like CAFs and tumor‐influenced T‐cell infiltration was able to be observed in this model.^85^ PDOs inclusive of autologous CD3^+^ and CD56^+^ TILs derived from lung adenocarcinoma, clear cell renal cell carcinoma and melanoma were able to be retained in a collagen gel matrix‐embedded air–liquid interface (ALI) cultures.^86^ Neal *et al*.^86^ demonstrated that PDOs derived from surgical resections (shown to retain both epithelial cell myofibroblasts and syngeneic TILs) could be isolated with the original T‐cell receptor spectrum preserved *in vitro*. Interestingly, myofibroblast populations decreased following passage in PDOs generated from kidney and colon tumors, a feature not noted in PDOs derived from NSCLC. Immunofluorescence and fluorescence‐activated cell sorting analysis showed that NSCLC, clear cell renal carcinoma and melanoma retained CD8^+^, CD4^+^ (T_h_) T cells, CD14^+^ or CD69^+^ macrophages, NK, NKT and B cells. Addition of IL‐2 was effective at preserving the endogenous TIL subsets, which had total life of approximately 1 month in organotypic media. Critically, anti‐PD‐1 monotherapy (nivolumab) was able to effectively activate the PD‐1‐expressing CD3^+^ T‐cell compartment and induce significant cytotoxicity in PDO cultures.^86^ In a feasibility study, Votanopoulous *et al*.^87^ described the ‘immune enhanced patient organoid’ model, a patient‐specific symbiotic co‐culture model pairing patient‐matched lymph node specimens or PBMCs with patient‐derived melanoma PDOs. Their hydrogel‐based system showed that embedding autologous tumor and lymph node cells together generated personalised immune‐competent PDOs that recapitulated clinical responses to ICIs targeting PD‐1 and CTLA‐4 in 6 of 7 patients.^87^ In our experience, lymph nodes are not commonly available in surgical specimens and represent a major limitation to the adoption of models such as these.

Co‐culturing with autologous peripheral blood cells enables the investigation of response of endogenous TILs. Dijkstra *et al*.^88^ demonstrated that by culturing PDOs with PBMCs, it is possible to induce the expansion of tumor‐reactive T cells. PDOs generated from mismatch repair‐deficient NSCLC and colorectal cancer cultured with autologous PBMCs resulted in a significant increase in CD8 expression in T‐cell populations.^88^ When combined with PD‐1 blockade, an increase in PDO‐induced IFN‐γ secretion was observed, indicating the usefulness of this platform for the examination of T‐cell‐mediated killing of cancer cells. As PDOs can be sustained for multiple weeks, it is evident from studies investigating PDOs derived from NSCLC and melanoma that each routine passaging varies the levels of stromal myofibroblasts, PDO TILs and expression of smooth muscle actin and vimentin further from the tumor original state.^89^ Whether this is common between all tumor types is something to expand upon. Furthermore, despite being more faithful models than cell lines, PDOs lack functional vasculature and it is likely that not all cell types of the TME are present in any particular organoid culture.^84^ Apart from CRC, standardisation or reproducibility of these models —including the composition of growth media and cytokine cocktails—has not been reached for most tumor types. The few studies to date have only incorporated pembrolizumab, nivolumab (anti‐PD‐1) and ipilimumab (CTLA‐4) therapy, and other ICIs or combination therapies with SOC therapies have only been described for feasibility studies in melanoma.^87^ Applications of cancer immunology in these models are becoming more popular as optimised methods and tools are increasingly published.

### Patient‐derived explants

Organotypic tumor explants are useful preclinical models to rapidly assess the physiological properties of primary tissues in an *ex vivo* setting.^90^ Explants are most easily generated from tissue obtained from surgical procedures. Tissue is then processed into slices, usually in the range of 100–400 μm, via manual processing or fragmentation using a device such as a vibratome or tissue chopper.^90^ Tissue explants can be directly cultured in a defined medium (which may contain autologous serum) with autologous components such as PBMCs and are typically fixed in formalin prior to paraffin embedding for subsequent histopathological and immunohistochemical analyses.^91^ Currently, the use of tumor tissue explants for immunotherapy testing is lacking; however, their potential usefulness in predicting clinical outcomes is becoming increasingly recognised. *Ex vivo* metastatic colorectal cancer tissue slices taken from a small cohort of patients demonstrated clinically relevant sensitivity to SOC agents and pembrolizumab (as measured by treatment‐induced cell death).^92^ Explants obtained from human primary breast cancer have also been evaluated for immunotherapy prediction.^93^ Transcriptomic analysis of breast cancer explants cultured with PD‐1, PD‐L1 and TIM‐3 inhibitors showed an upregulation in expression of pathways pertaining to antitumor immune response.^93^ These data also revealed that the blockade of some immune checkpoints can induce upregulation of others as a compensatory measure (including of *ICOS*, *CTLA4*, *BTLA* and *OX40L* in response to anti‐PD‐L1 and anti‐TIM3 treatment),^93^ suggesting that the explant culture technique may be used to test the sequential combination of immunotherapies and other SOC therapies. NSCLC tissue slices cultured with autologous PBMCs reveal a revitalised activation of innate immunity pathways.^94^ Overall, the utility of explants as a tool for precision immunotherapy is in its infancy but warrants deeper investigation. Preserving T‐cell function for an extended period of time continues to be a challenge, with studies indicating a rapid drop in CD3^+^ cell viability after about 3–4 weeks.^95^ Tissue slices have not been used widely for immunophenotyping and immune checkpoint discovery beyond murine models and thus represent an opportunity to incorporate clinical tumor samples in this functional testing platform.

### Emerging preclinical immunotherapy prediction models

Microfluidics‐based ‘organ‐on‐a‐chip’ (also known as microphysiological systems) are favored next‐generation platforms for modelling drug response in an *in vitro* environment. Such systems may be precisely manipulated to better recapitulate the spatial location of the cells, vascular regions and biophysical forces in the TME (Figure 3). Additionally, important factors for immuno‐oncological investigations such as chemokine and oxygen rates can be precisely controlled. These chips are most commonly composed of polydimethylsiloxane (PDMS) and glass and contain many microchannels and compartments that can be subject to minute and accurate fluidic and biochemical adjustments.^96^ The control over these parameters, alongside design choices in channel architecture and cellular contents, allows for many organs and niches of the human body to be recapitulated to a potentially high degree of accuracy. The transparent substrates used also have an ideal optical index that is advantageous for standard microscopy imaging. In an immuno‐oncology setting, organs‐on‐a‐chip have been used to model anti‐PD‐1/PD‐L1 therapy in a small number of malignancies.^97^ Cytokine profiling of metastatic melanoma patient‐derived xenografts (PDXs) and PDOs combined with autologous myeloid and lymphoid populations in a short‐term 3D microfluidic culture has demonstrated the enhancement of response to anti‐PD‐1 when co‐treated with anti‐TBK1/IKKε inhibitors.^98^ Furthermore, Cui *et al*.^99^ described a patient‐derived ‘glioblastoma‐on‐a‐chip’ model to assess inhibition of PD‐L1 and CSF‐1R across various molecular subtypes of glioblastoma (GBM). It was discovered through this platform that in comparison with proneural GBM, glioblastomas affecting the mesenchymal niche attracted significantly higher levels of CD163^+^ M2 TAMs and PD‐1/PD‐L1 and responded better to ICI treatment.^99^ The organ‐on‐a‐chip model is yet to fully realise its translational and clinical potential; however, initial promising results, combined with informed chip and assay optimisation, will undoubtedly lead to a greater impact in future.^97^

### Towards the integration of immunotherapy co‐culture precision medicine tools

**Figure 4:**
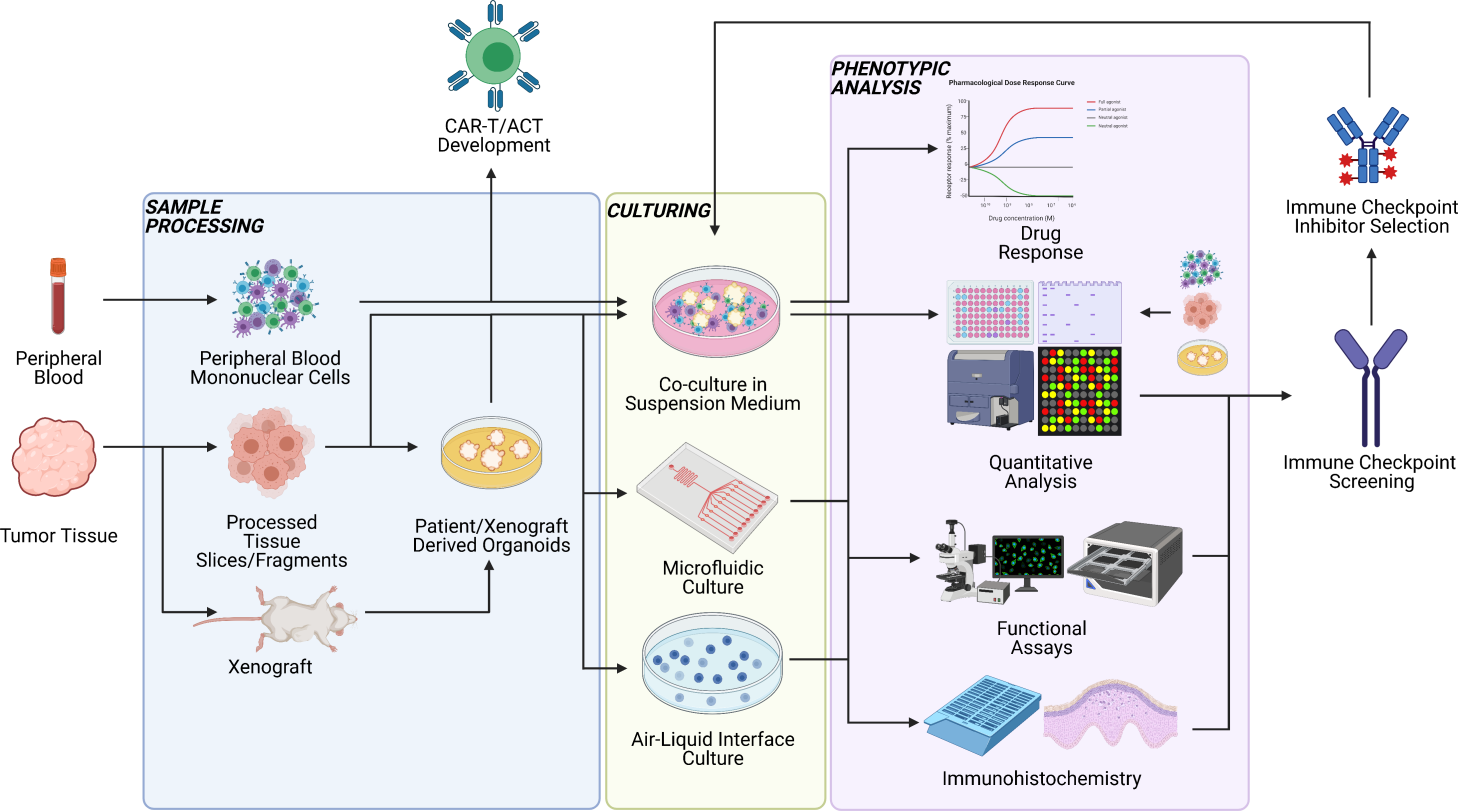
Summarised workflow for immunological analysis of patient material in cancer. Tumor tissue is processed into PDOs, PDXs or slices, and whole peripheral blood is harvested for PBMC extraction. These autologous components are then cultured together, commonly in suspension, at the air–liquid interface or processed into a microfluidic chip. Addition of anticancer drugs followed by systemic functional and quantitative analysis allows for the identification and prevalence of clinically relevant immune checkpoints and a predictive examination of the efficacy of treatment. ACT, adoptive cell transfer; CAR‐T, chimeric antigen receptor T cell. This figure was created with Biorender.com.

By capturing individual patient diversity, 3D models that also retain TME elements provide platforms to explore patient drug responses in clinical settings where immunotherapy is an option. Such an assay would be used alongside current clinical, biomarker‐supported therapies, and within a clinically relevant time frame. However, the translation of these model systems into clinical settings is complex. As discussed above, each personalised model has benefits that may provide important information as a companion diagnostic assay during a patient’s clinical management. Immune‐retaining PDOs and novel bioengineered constructs could potentially serve as important tools to direct treatment choice at the bedside, following tumor recurrence and ancillary to clinical trials. Furthermore, in the setting of ineligibility for chemotherapy or unresectable tumors, it is paramount that patient‐derived immune‐encompassing models are developed towards clinical utility to help direct immunotherapy alongside ICI pathology screening (Figure 4). To instruct patient care, a number of significant hurdles will need to be addressed, including the standardisation and cost of patient‐derived immune co‐cultures (i.e. media formulations and use of biological and synthetic matrices), optimised clinical workflows to utilise surgical resections (i.e. ethics and clinical partnerships with industry), increasing the establishment rates (i.e. increasing PDO success rates) and refinements on the speed and quality of sample processing pipelines to culture‐to‐drug treatment workflows (Figure 4). Additionally, a lack of standardised quality control and drug treatment endpoint analysis methods currently prevent integration into the clinical space. As more studies incorporate TME components, producing larger‐scale and higher‐resolution data, improvements in automation and context‐driven mathematical modelling of the tissue milieu will help address these key significant hurdles and will allow randomised controlled trials to evaluate the usefulness of 3D models as clinical tools. In the coming years, the development of novel technologies and refinement of the culture parameters for these models have the potential to impact immunotherapy rationalisation, and thus the approach to treating cancer and the way patient care is personalised (Table 3).

**Table 3:** Personalised immune‐based drug screening platforms

Model type	Co‐culture		Technology	Prestimulation	ICIs used	Analysis method	Time frame	Comments	References
	Malignancy	Immune cells (E:T)							
*Ex vivo*/2D	NSCLC (LLC), CRC (CT26)	CD8+ T cells	Cell culture flask	Nil	αPD‐L1 (*in vivo*)	Luciferase, CD107 degranulation, flow cytometry	2 days *ex vivo*	Autologous	Pimentel *et al*.^100^
2D	Prostate (PC‐3) and breast (MCF7)	PBMC, NK (NK92), T cell (TALL‐104), B cell (Raji), CAR‐T (various E:T)	xCELLigence RTCA system	IL‐2	αPD‐1	Cellular impedance changes in cellular attachment strength, flow cytometry	70 h	Allogeneic	Cerignoli *et al*.^78^
2D	Breast, lung, liver	Jurkat, PBMC	Cell culture flask	αCD3, αCD28	αPD‐1, αPD‐L1	RT‐qPCR, flow cytometry, immunoblot	2 days	Allogeneic	Zheng *et al*.^77^
Spheroid	CRC (HT‐29), PDAC (PSN‐1), GBM (U251MG), lung (H1299)	T cells, NK (10:1)	ULA plates	Nil	αPD‐1, αPD‐L1	IHC, Luciferase (luminescence), flow cytometry	4 days	Allogeneic	Zboralski *et al*.^101^
Spheroid	CRC (LS174T, LoVo)	PBMC (10:1)	Hanging drop	IL‐2, IgG‐IL‐2v	Nil	IHC, cytometric bead array, flow cytometry	4 days	Allogeneic	Herter *et al*.^102^
Spheroid	CRC (HT‐29, DLD1)	T cells, NK (10:1)	ULA plates	IL‐15, IL‐7	Nil	Flow cytometry, bright‐field and fluorescent microscopy (spheroid volume and caspase 3/7 staining)	6–7 days	Allogeneic	Courau *et al*.^103^
Spheroid	Breast (MDA‐MB‐231)	PBMC (1:1)	Scaffold free, liquid overlay	PMA/Ionomycin	Nil	Brightfield microscopy (spheroid area), CFSE and CellTracker™ Orange CMTMR, flow cytometry, ELISA (IFN‐ϒ)	10 days	Allogeneic	Saraiva *et al*.^104^
PDO	PDAC	CD3^+^ T cells (1:1)	Matrigel	αCD3, αCD28	Nil	Flow cytometry, immunofluorescence	6 days	Autologous	Tsai *et al*.^85^
PDO	Melanoma	PBMC and lymph node cells (1:1)	HA/collagen‐based hydrogel	Nil	αPD‐1 (pembrolizumab) αCTLA‐4 (ipilimumab)	LIVE/DEAD (Thermo Fisher), immunofluorescence, CellTiter‐Glo 3D	10 days	Autologous	Votanopoulos *et al*.^87^
PDO	NSCLC, CRC	CD8^+^ T‐cell (5:1)	Matrix scaffold (Geltrex)	IFN‐ϒ, IL‐2, anti‐CD28	αPD‐1	Live‐cell imaging (CellTrace Far Red), flow cytometry	14 days	Autologous	Cattaneo *et al*.^105^
PDO	CRC, NSCLC	PBMC (20:1)	Matrix scaffold (Geltrex)	IFN‐ϒ, IL‐2, anti‐CD28	αPD‐1	Flow cytometry, fluorescent microscopy (CellTrace Yellow, Far Red, caspase‐3/7 probe), IHC, Live imaging	14 days	Autologous	Dijkstra *et al*.^88^
PDO	ccRCC, NSCLC, melanoma, bladder	*In situ* TILs	ALI technique (collagen gel matrix embedded into membrane)	IL‐2, αCD3, αCD28	αPD‐1 (nivolumab)	Flow cytometry, qRT‐PCR, IHC	7 days	Autologous	Neal *et al*.^86^
PDO	Chordoma	*In situ* TILs (CD8^+^ T cells)	2% Matrigel‐coated microchambers	Nil	αPD‐1 (nivolumab)	Fluorescence imaging (% DAPI‐stained cells)	24 h	Autologous	Scognamiglio *et al*.^106^
Tissue explant	Breast	*In situ* TILs	Cryopreserved ~2‐ to 4‐mm manually sliced explants in 6‐well tissue culture plates	IL‐2	αPD‐1 (pembrolizumab), αPD‐L1 (atezolizumab), αTIM3	Flow cytometry	9 days	Autologous	Saleh *et al*.^93^
organotypic slice culture	PDAC	Nil	250‐μm‐thick slice, PTFE membrane	Nil	αPD‐1 (nivolumab)	Live microscopy, flow cytometry, cytokine quantification (Luminex assay)	2–6 days	Autologous	Seo *et al*.^107^
3D microfluidic culture	HNSCC	PBMC (NA)	3D PDMS microfluidic chip	Nil	αPD‐L1, αIDO 1	fluorescent microscopy (CellTrace Far Red, Violet) for enumeration	3 days	Allogeneic	Al‐Samadi *et al*.^108^
3D microfluidic culture/ 2D microfluidic culture	HeLa cells	NK (NK‐92) (NA)	Injection‐moulded microfluidic device, cell culture flask	Nil	Nil	fluorescent microscopy (CSFE, Far Red), live imaging	24 h	Allogeneic	Park *et al*.^109^
3D microfluidic culture	HGSC, NSCLC	*In situ* TILs	COP microfluidic device design	Nil	αPD‐1 (pembrolizumab), αCTLA‐4 (ipilimumab)	Immunofluorescence, cytokine ELISA	5–9 days (*ex vivo*)	Autologous	Aref *et al*.^110^

2D, two‐dimensional; 3D, three‐dimensional; ALI, air–liquid interface; CAR‐T, chimeric antigen receptor T cells; ccRCC, clear cell renal cell carcinoma; CD, cluster of differentiation; CMTMR, 5‐(and‐6)‐(((4‐chloromethyl)benzoyl)amino)tetramethylrhodamine; COP, cyclic olefin polymer; CRC, colorectal cancer; CSFE, carboxyfluorescein succinimidyl ester; CTLA‐4, cytotoxic T‐lymphocyte‐associated protein 4; DAPI, 4′,6‐diamidino‐2‐phenylindole; ELISA, enzyme‐linked immunosorbent assay; E:T, effector‐to‐target ratio; GBM, glioblastoma; HGSC, high‐grade serous carcinoma; ICI, immune checkpoint inhibitor; IDO1, indoleamine 2,3‐dioxygenase 1; IgG, immunoglobulin G; IHC, immunohistochemistry; IFN‐ϒ, interferon‐gamma; IL‐2, interleukin‐2; NA, not available in study; NK, natural killer; NSCLC, non‐small‐cell lung cancer; PBMC, peripheral blood mononuclear cell; PCa, prostate cancer; PD‐1, programmed death‐1; PDAC, pancreatic ductal adenocarcinoma; PD‐L1, programmed death‐ligand 1; PDMS, polydimethylsiloxane; PMA, phorbol 12‐myristate 13‐acetate; PTFE, polytetrafluoroethylene; RTCA, real‐time cell analysis; RT‐qPCR, reverse transcription–quantitative real‐time polymerase chain reaction; TILs, tissue‐infiltrating lymphocytes; TIM3, T‐cell immunoglobulin mucin‐3; ULA, ultra‐low attachment.

## Concluding remarks

The landscape of 3D tissue recapitulation is in a state of rapid innovation and optimisation and has provided translational immuno‐oncology with novel platforms for therapeutic screening. In addition, the theory of culturing tumor‐derived material with interfacing components of the TME niche has great potential to advance the personalisation of effective anticancer treatment. Short‐term immuno‐oncological co‐cultures have been fruitful, but long‐term modelling continues to be a challenging prospect. Issues surrounding viability of both the tumor model and immune cells (or other autologous cellular components) become increasingly prominent with time. It is important that efforts to increase viability of these components alter phenotypic properties of the recapitulated TME as little as possible. Cytokines such as IL‐2, IL‐6 and GM‐CSF can be incorporated for the preservation and expansion of immune cells, and replenishing culture media will offer a degree of protection from non‐specific cell death; however, these will require further validation of robustness in the long term. It is also evident that a greater majority of studies focus on T‐cell‐specific immune checkpoint interactions despite incorporating a wider population of immune cells. Further investigation is required on the therapeutic implication on the immune checkpoints of myeloid‐derived cells and non‐T‐lymphoid cells (NKs) in co‐culture. Beyond this, co‐cultures could potentially be used to rapidly compare immunological responses between multiple metastatic sites in individual patients within a contained setting. Organ‐on‐a‐chip models also have significant potential to model the cancer–immune axis beyond the TME and examine aspects such as the contribution of lymphatic and mesenchymal components. Continued optimisation of 3D immunological co‐culture techniques will influence the landscape of translational precision immunotherapy, enabling new discoveries and providing opportunities to develop personalised, functional testing platforms that can predict treatment efficacy to improve patient outcomes.

## Author contributions

**Nathan Mackenzie:** Conceptualization; investigation; visualization; writing – original draft; writing – review and editing. **Clarissa Nicholls:** Writing – review and editing. **Abby Templeton:** Writing – review and editing. **Mahasha Perera:** Writing – review and editing. **Penny Jeffery:** Writing – review and editing. **Kate Zimmermann:** Writing – review and editing. **Arutha Kulasinghe:** Writing – review and editing. **Tony Kenna:** Conceptualization; writing – original draft; writing – review and editing. **Ian Vela:** Conceptualization; funding acquisition; project administration; resources; supervision; writing – review and editing. **Elizabeth Williams:** Conceptualization; funding acquisition; project administration; resources; supervision; writing – review and editing. **Patrick Thomas:** Conceptualization; funding acquisition; project administration; resources; supervision; writing – original draft; writing – review and editing.

## Conflicts of interest

The authors declare no conflict of interest.
